# Childcare Physical Activity Interventions: A Discussion of Similarities and Differences and Trends, Issues, and Recommendations

**DOI:** 10.3390/ijerph16234836

**Published:** 2019-12-02

**Authors:** Rachel A. Jones, Eduarda Sousa-Sá, Michele Peden, Anthony D. Okely

**Affiliations:** Early Start, University of Wollongong, Northfields Ave, Wollongong, NSW 2522, Australia; emdsr885@uowmail.edu.au (E.S.-S.); mep948@uowmail.edu.au (M.P.); tokely@uow.edu.au (A.D.O.)

**Keywords:** physical activity, early childhood education and care settings, professional development, interventions, preschool children

## Abstract

Early childhood education and care (ECEC) settings have a pivotal role in the promotion of physical activity for young children, and thus, the number of ECEC-based physical activity interventions has exponentially increased in the last two decades. The aim of this study was three-fold: (1) to discuss some of the similarities and differences in ECEC-based physical activity interventions, (2) to highlight current trends and issues in the ECEC sector relating to such interventions, and (3) to provide recommendations for future interventions. Twenty-four individual studies are discussed. Most studies have targeted children aged between 3 and 5 years and involved children participating in additional physical activity opportunities while at childcare. In all studies, educators participated in some professional development either prior or during the intervention. Less the half of the studies discussed reported significant positive changes in physical activity outcomes. Those involved in developing future interventions will need to consider current national and international trends in the ECEC sector (e.g., over-crowded curriculum, administrative requirements, and more highly-qualified educators devoting time for business development), as well as creative and unique ways of delivering ECEC-based physical activity interventions.

## 1. Introduction

Healthy physical activity habits need to be established from a young age to ensure optimal health, well-being, and development [1]. Furthermore, the promotion of physical activity should start as early as possible, as physical activity levels track from early childhood to childhood and adolescence [2]. The early childhood education and care (ECEC) setting has been identified as a critical setting for the promotion of physical activity and physical activity-related behaviors [3]. The ECEC setting is critical in the promotion of these behaviors, because many children across the globe spend more awake time in these settings compared to other settings. In some countries, children spend seven to eight hours in such settings, five days a week [4].

Current data suggests that approximately 50% of children whilst attending ECEC do not meet the recommended levels of physical activity (15 min/h for every hour of care), and spend up to 50% of their time in sedentary activities [5]. To combat these less-than-optimal trends in physical activity, the number of ECEC-based physical activity interventions have steadily increased over the past two decades. Consequently, a number of systematic reviews have been published. These reviews have varied in focus, number, and type of studies and analyses. For example, Engel et al. [6] reported on studies that had evaluated the relationship between fundamental motor skills and physical activity, whilst Timmons et al. [7] included studies that reported physical activity and health outcomes. Ward, et al. [8] included studies that investigated the relationship between educator behaviors and physical activity. Gordon et al. [9] and Finch et al.’s [10] reviews were both inclusive of meta-analyses.

These reviews provide some high-level recommendations for future ECEC-based interventions, (e.g., reporting of cost data and consideration of sustainability [10,11]); however, they generally do not provide recommendations in light of current trends and issues within ECEC sector. Therefore, this discussion piece was completed with the aim of providing a high-level overview of some of the key ECEC-based physical activity interventions, and in turn, highlighting future recommendations for such interventions in light of the current trends and issues within the ECEC sector. This discussion aimed to provide a useable and accessible document that provides realistic and innovative recommendations for future practice.

## 2. Materials and Methods

The studies discussed were sourced from 10 systematic reviews, published between 2009 and 2018 [6,7,8,9,10,11,12,13,14,15]. These studies broadly represent the current evidence and meet the following four criterium: (1) focused on physical activity exclusively, or had a physical activity component (i.e., those with a focus on obesity prevention); (2) were delivered in an ECEC setting; (3) were a randomized controlled trial (RCTs), pilot RCTs, cluster RCTs, or quasi-experimental trials; (4) assessed physical activity either subjectively or objectively, and reported physical activity intensity was chosen to be included in the discussion paper. Data such as sample size, length of intervention, intervention components, facilitators of the interventions, type of professional development delivered, instruments used to measure physical activity, and physical activity outcomes were extracted to provide an overall picture of ECEC interventions within the current literature, and to enable discussion pertaining to similarities and differences. The studies included within this paper were not exhaustive, but rather representative of the current evidence. Data from studies were extracted by three researchers (R.A.J., E.S., and M.P.).

## 3. Results

The results section highlights some of the similarities and differences between the identified studies. The study characteristics of the 24 studies identified are highlighted in Table 1. Eighteen studies (75%) had physical activity as the primary outcome [16,17,18,19,20,21,22,23,24,25,26,27,28,29,30,31,32,33], and six (29%) had physical activity as a secondary outcome (i.e., the main outcome was another outcome—for example, weight-related outcomes) [34,35,36,37,38,39]. Studies were conducted in a number of different countries, including the United States (*n* = 10) [16,17,18,19,20,30,33,35,36,37], Switzerland (*n* = 2) [21,31], Belgium (*n* = 2) [22,24], Germany (*n* = 2) [23,24], Israel (*n* = 2) [34,38], Australia (*n* = 3) [25,27,28], Canada (*n* = 1) [26] and the United Kingdom (*n* = 2) [29,39]. Most studies targeted children aged between three and five years. All but three studies recruited children from non-specialized populations [16,19,35,36]. The sample size ranged from 21 to 826 participants, with six studies recruiting less than 100 participants at baseline [16,17,18,26,27,30].

The interventions ranged from four weeks to one year (pre-test to post-test), and a nearly a third of the studies (7/24, 29%) included a follow-up period (i.e., data were collected at three time points: pre-test, post-test, and follow-up) [24,29,31,35,36,38,39]. Three studies included a follow-up time point beyond one year [35,36,38].

In the majority of interventions, educators were encouraged to provide additional time for children to spend in physical activity (either structured or unstructured physical activity learning experiences). In most studies, children were encouraged to spend between 20 and 45 min in additional physical activity, two or three times a week. A small number of studies involved modifications to the indoor environment only [24], the indoor and outdoor environments [21,25], or the outdoor environment only [22]. One study provided funding to the intervention centers for the purchase of the mobile equipment and for the rearrangement of the environments to make them more activity-friendly (no further details provided) [21].

Most (87%) interventions were facilitated by educators. A small number of interventions were co-led by researchers and educators. For example, Eliakim et al.’s [34] intervention was led by a professional youth coach for two days, and then by the educators for the other days. Similarly, the study by Nemet et al. [38] was led by a professional youth coach once a week and the educator on the other days. Alhassan et al.’s [18] intervention was exclusively facilitated by researchers, although it was helped by the educators, and De Bock et al.’s [23] study was led exclusively by external gym trainers. In general, educators received professional development in the aims of the intervention and the intervention components prior to the start of the intervention. All professional development was facilitated face-to-face; however, the intensity and frequency of the training and the resources provided varied. For example, Alhassan et al. [18], Bonvin et al. [21], and Finch et al. [25] included four to eight hours of educator professional development. Various resources were provided as part of the professional development, such as prepared lesson plans or resources (e.g., newsletters, posters, music CD, stickers, child achievement cards) [18,19,20,24], equipment [17,18,24,31], and additional face-to-face support [21,25,31].

The majority of studies used accelerometers to assess physical activity, which were worn for a number of hours or days [16,17,18,19,20,21,22,23,24,26,27,28,29,30,31,32,33,37,38,39]. Cut points used to categorize physical activity were “Pate” [40] and “Sirard” [41] references. Other instruments used to measure physical activity included pedometers (steps/day) [25,34], heart rate monitors [30], and parent-proxy report [34,35].

Of the 18 studies with physical activity as the main outcome, eight (44%) reported significant changes in physical activity outcomes at the end of the intervention [17,19,23,24,26,30,31,33]. Significant changes in moderate- to vigorous-intensity physical activity (MVPA) and vigorous-intensity physical activity (VPA) were reported for Annesi et al. [19], De Craemer et al. [24] and Trost et al. [33]. Significant changes in total physical activity was reported for Alhassan et al. [17] and De Bock et al. [23]. Puder et al. [31] reported significant changes in fitness levels between the intervention group and the control group. Of the six studies that included physical activity as a secondary outcome, half (50%) reported significant changes in physical activity levels [34,37,38]. Fitzgibbon et al. [37] reported significant changes for MVPA and VPA between the intervention group and the control group, whereas Nemet et al. [38] and Eliakim et al. [34] reported significant changes in the number of steps and fitness levels, respectively.

## 4. Discussion

This paper aimed to provide a broad sweeping picture of previous ECEC-based physical activity interventions and highlight some similarities and differences between studies. On the whole, there were very few consistent characteristics between the studies, with studies varying in design, sample size, and length of intervention. However, two characteristics were consistent in all interventions: (1) the provision of additional time for physical activity for children each week, and (2) the participation of educators in professional development. Given the consistency of these characteristics, innovative, creative, and engaging mechanisms for ensuring that these components are prioritized in future interventions may be of benefit.

Three main research gaps were identified: (1) the lack of interventions in infants and toddlers; (2) professional development delivery methods (e.g., face-to-face, online, blended); and (3) the need for longer-term assessments. Despite physical activity being an important component of holistic child development and a behavior that should be encouraged from birth [1,42], all but one study had a mean participant age between 3–5 years [30]. This may be due to educators and researchers thinking that physical activity learning experiences are more relevant for older children, as these children have increased movement and cognitive abilities when compared to children aged 0–3 years. Despite this, it is still critically important programs for children aged 0–3 years provide a balance of child-initiated and adult-led experiences (all of which constitutes a play-based curriculum) [43]. Such interventions would need to be age- and developmentally-appropriate, and thus may “look” different from those implemented for older children. For example, the inclusion of “tummy time” and crawling activities would be a key component in interventions targeting younger children.

Professional development received by educators in all ECEC-based interventions reviewed was facilitated face-to-face. This type of professional development is widely used within the ECEC sector; however, it is associated with a number of limitations. For example, the limited transfer of knowledge [44] and a “top-down approach”, which often results in not meeting the needs and wants of educators [45,46,47,48]. Alternate methods of professional development, such as online professional development, mentoring, coaching, and blended (involving both face-to-face and online components) professional development may be more viable for the ECEC sector. To date, only one study comprising blended professional development for the ECEC sector has been evaluated: positive changes in child-level data and center-level physical activity data were reported [49]. This method of delivery seems to hold promise for the ECEC sector; however, additional studies are needed to support these initial findings [49].

Only six studies assessed physical activity outcomes at one year [24,31,32,35,36,38,39], and only four studies assessed physical activity outcomes more than one year [31,35,36,38]. Longer follow-up periods are recommended, as adequate “soak time” is needed for change to become part of the ECEC routine, and for behaviors to change and new norms to be re-established [50]. Educators need the time to process, discuss, and reflect on the new information offered in professional development sessions. Additionally, they need time to critically reflect on current practices, and devise sustainable methods of change within their centers, with their staff that are consistent with their pedagogical practices [51]. The children also need time to adapt to new routines and learning experiences, and to become comfortable with these experiences. Change within the ECEC sector is no different from other educational sectors; it is typically met by educators with resistance and considerable effort, therefore support is needed to ensure that long-term changes are implemented and sustained [51]. The best method of support to enable long-term change in ECEC settings remains unknown; however, a recent study suggested that telephone support or ongoing mentoring programs that involve site visits may be the most preferred and successful option for the ECEC sector, at least in high-income countries like Australia [52]. Additional research is needed to determine the best support mechanisms for the ECEC sector that will ensure that change in children’s physical activity behaviors are maintained.

### 4.1. Trends

The ECEC environment has changed significantly since 2000, as new national and international regulations have been introduced, making it a complex environment for the implementation of interventions [43]. As new regulations have been enforced, the demands on educators have exponentially increased, with increased documentation required and shifts in job description for senior staff (i.e., higher-qualified educators are now devoting time to business development rather than teaching and mentoring) [53,54].

Given the complexity and uniqueness of the ECEC environment [54], several additional trends may need to be considered in the formative stages of future interventions. These trends are not explicitly discussed in the papers reviewed (see Results section), but are based on current evidence and the expert opinion of researchers and practitioners working in the sector internationally over a number of years. ECEC environments, at least within the Australian context, have an exceptionally high turnover of staff, which makes transfer of knowledge and consistent pedagogical practices difficult [51]. Future ECEC-based physical activity interventions need to consider how to maximize knowledge transfer during the implementation period (and beyond) of an intervention. Ongoing and online professional development may be one viable method of ensuring consistent, high-quality pedagogical practice [49].

Shaping or reshaping educators’ perceptions in relation to physical activity may also be an important area to investigate in future interventions. It is well-established that educators have a critical role in shaping the behaviors and patterns of the children in their care [55]. Some studies have shown that educator’s behaviors positively influence physical activity behaviors of children [3,56,57]. That is, where educators provide positive prompts in relation to physical activity activities or role model positive physical-activity behaviors, children are more consistently more active [56,57]. In contrast, and perhaps more common, other studies suggest that the main role of educators in relation to physical activity is a passive supervisory role, and that young children are sufficiently active during ECEC hours and do not need intentional physical activity learning experiences [5,58]. Educators also shy away from providing intentional physical activity experiences, due to their own low levels of self-efficacy in this area [59]. Several studies have highlighted that educators do not feel comfortable or knowledgeable enough about physical activity or know what experiences to offer the children, therefore tend not to offer any [59,60]. Allocating time to inform educators of the vital importance of physical activity in children’s development and discussing examples of successful and engaging physical activity learning experiences may be a critical component of ECEC-based interventions that focus on physical activity or are inclusive of a physical activity component. Physical activity for young children is often (mis)conceptualized by educators as structured activities or activities that just involve running or jumping at high intensity. However, for preschool children, physical activity is a broad term that should be inclusive of risky play; fine and gross motor skills; social interactions; movement vocabulary; and engagement and communication between children, their families, and educators [61]. It is much more than the traditional perceptions of physical activity.

Physical activity is often underrepresented in both curriculums and current practice. Other key learning domains, such as the cognitive and social/emotional domains, seem to take higher priority. This may be attributed to the greater diagnosed or undiagnosed needs of children in ECEC settings, where children with higher social and emotional needs require more attention from educators, and thus more time is spent specifically on enhancing the social and emotional needs of children. Certainly within the Australian environment, there is a push for heightened child agency (i.e., supporting children’s rights within the learning environment), optimizing child self-regulation and supporting children affected by trauma—aspects that align within the social and emotional domains, therefore offering limited time for other domains, such as the physical [43]. The reduced emphasis on the physical learning domain may also be a consequence of the requirements for educators to provide holistic comprehensive programs. For example, educators are required to integrate science, technology, engineering, and mathematics (STEM) learning experiences, as well as learning experiences that are inclusive of sustainability and cultural competence components, health and hygiene, and child protection [43]. In some countries, the overarching push for children to become “school-ready” well before they enter school [62] may also influence the time spent in high-quality physical activity learning experiences. In such countries, the emphasis is on learning to read and write to ensure that children are adeptly prepared to transition to formal schooling [62]. However, school readiness is actually about the ability for children to develop independence, follow instructions, form friendships and socialize, listen, complete an experience or task, communicate, and engage and interact with their peers and educators [63,64]. It is also about the appropriate development of fine and gross motor skills and strong core muscle development. Many of these attributes are developed through the participation of age-appropriate, physically active, inclusive intentional learning experiences rather than academic-based skills [63,64].

The administrative burden of educators has exponentially increased in the past decade [54]. Whilst educators have always been required to provide documentation on their educational programs and the developmental outcomes of children, they are now also required to adhere to comprehensive assessment and compliance requirements [54]. For example, in Australia, ECECs must gather evidence and complete documentation across seven quality areas outlined under the National Quality Standards [43], which involves completing an ongoing Quality Improvement Plan and providing evidence of how educators are critically reflecting on policies and practices across the afore-mentioned areas. Furthermore, in many countries, educators are required to be in constant communication with parents throughout the day via daily electronic notice boards (e.g., Kinderloop (https://kinderloop.com)) so that parents can view the learning experiences their children participate in throughout the day [65]. These added responsibilities now have the potential to deter educators away from their core business, which is teaching children [65].

### 4.2. Recommendations

In light of the studies discussed, and considering the gaps in the current evidence and the current trends within the international ECEC sector, future ECEC-based physical activity interventions could consider the following four recommendations. Recommendations 1 and 2 are based on data extracted from the papers discussed in the Results section, and recommendations 3 and 4 are based on current evidence from broader ECEC literature, which is applicable to ECEC-based physical activity interventions. Future ECEC-based interventions could consider:

(1) The provision of high-quality professional development for educators prior, during, and beyond the intervention phase. Given the low competence and confidence levels of most educators in the area of physical activity, explicit professional development should be a priority. The professional development should be delivered by experts with experience working within the ECEC sector, and should include information on the critical role of the physical domain in child development. Furthermore, providing additional training to pre-service educators in the area of the physical domain is important.

(2) Interventions that are “outside the box”. The majority of ECEC-based physical activity interventions have been standard in intervention design and delivery; they have involved face-to-face professional development, a prescriptive physical activity component for the children, and a few suggestions of additional resources. Future interventions should start to think outside the box with regard to intervention components and delivery. The following questions might be helpful as future interventions are planned: Can blended professional development be used? What role can technology play in physical activity interventions? Is it important to consider the health, well-being, and physical activity levels of the educators? Can interventions target educators’ physical activity? Can interventions incorporate a combination of intervention components—for example, structured physical activity learning experiences, outdoor marking, and energy breaks? How can physical activity be integrated into all aspects of the normal routine? How can physical activity experiences be extended, expanded, or enhanced [66] in ECEC settings?

(3) Meaningful and trustworthy collaborations between educators and researchers. Developing collaborative professional relationships between researchers and educators can help maximize children’s physical activity outcomes. A recent paper suggests the successful collaboration involves researchers spending time learning about the ECEC environment and presenting research findings in an easily interpretable manner to educators [59], thereby ensuring that research is translated into relatable practical strategies that enable educators to critically reflect on current practices. Furthermore, the importance of researchers understanding the profound place of relationships within the ECEC sector and the importance of including educators in the research process were also highlighted as being critical [59]. Trustworthy and meaningful relationships between educators and researchers are particularly paramount for the success of intervention studies, which often require additional responsibilities from educators and change within their centers [59].

(4) The influence of the quality of the ECEC environment. It is well-established that the quality of the ECEC environment has a significant influence on children’s outcomes, both in the short and long term [67]. Several key studies, such as the Effective Pre-school, Primary and Secondary Education Project [67], have shown that higher quality ECEC environments results in better cognitive, social, and behavioral outcomes, immediately (ages 3–5 years) and several years later (e.g., at ages 11 and 16) [55]. In fact, literacy outcomes at age 11 are influenced more by the quality of the ECEC environment than the family’s income, father’s education, or the quality of the primary school [55]. Thus, future interventions should consider assessment of the quality of the environment in relation to physical activity, and consider how this could be improved through intervention components. To date, there are relatively few reliable and valid instruments available that assess the quality of the ECEC environment in relation to physical activity. The MOVERS Scale [61], which was published in 2017, is the first instrument to assess the product and process quality of the ECEC environment in relation to physical activity. It has four sub-scales: curriculum, environment, and resources for physical development; pedagogy for physical development; supporting physical activity and critical thinking; and parents/guardians and staff [61]. The MOVERS Scale focuses on the influence of the educators with regard to physical activity, as well as physical activity learning experiences offered to the children. High-quality instruments that measure the quality of the ECEC environment in relation to physical activity should be considered as a measure in future interventions.

This discussion provides a broad summary of ECEC-based physical activity interventions, and highlights some of the similarities and differences between key studies. The discussion and recommendations should be considered in light of the that fact that the studies discussed were not exhaustive, but rather a representative sample of the current evidence. Single-group pre-test/post-test studies, studies specific to family day care, or those that focused on adherence to policies [68] were not included. Lessons and recommendations can also be ascertained from such studies. A formal review of reviews was not conducted. A formal review of reviews would have provided further evidence of pooled data in relation to successful interventions, and would have provided information pertaining to the methodological quality of the studies.

To date, broad sweeping recommendations for future ECEC-based interventions have been provided. For example, future interventions should consider longer-term follow-up, multi-strategy interventions that include changes in the physical activity environment, reporting of cost-effectiveness, and the consideration of sustainability [10,11]. Future ECEC-based interventions are certainly important to contemplate, but perhaps a broader view also needs to be considered. Perhaps time needs to be spent advocating for additional focus of the physical domain in training programs (such as undergraduate degrees, diplomas, and certificate training). How can policy be influenced to highlight the importance of the physical domain, physical development, and physical activity for young children? How can national training bodies (e.g., the Early Childhood Training and Resource Centre in Australia) be encouraged to prioritize (or at least include) professional development in this area? What intervention strategies can be translated into policy and practice, and be rolled out throughout countries?

With the release of recent physical activity guidelines for the early years from a number of countries (including Australia, Canada, the United Kingdom, and South Africa [69,70,71,72]), as well as those released from the World Health Organization [73], it is clear that the promotion of physical activity must continue to be a global priority to ensure the health and wellbeing of young children now and in the future. The ECEC environment, although challenging and complex, needs to continue to be a key environment for the promotion of physical activity. ECEC-based physical activity interventions should continue to be an important area of focus and funding simultaneously with broader advocacy approaches.

## 5. Conclusions

Physical activity promotion and participation is important from a young age for optimum health and wellbeing. ECECs have a critical role to play in the promotion of physical activity behaviors; however, during the design phase of future interventions the current issues and trends of ECECs need to be seriously considered in order for effective ECEC-based physical activity interventions to be successful. Interventions, which are sustainable and transferable, should be a priority.

## Figures and Tables

**Table 1:** Study characteristics included in the overview of reviews.

Reference and Country	Design, Sample at Baseline	Length and Follow-Up	Intervention	PA Outcome/PA Measurement	PA Findings
Alhassan et al., 2007 United States [16]	Pilot RCT, 32 (3.6 yrs)	3 mths	2 × 30 min outdoor play sessions per day, 2/wk	Primary/ Accelerometers	No significant difference in PA at 3 mths
Alhassan et al., 2012 United States [17]	RCT, 71 (3–5 yrs)	6 mths	Daily 30 min structured lessons focusing on locomotor and movement skills	Primary/ Accelerometers	Significant difference in TPA and SB during preschool hours at 6 mths
Alhassan et al., 2013 United States [18]	Pilot cluster RCT, 67 (3–5 yrs)	4 wks	30 min outdoor structured curriculum based lesson, 3 times/wk during play time	Primary/ Accelerometers	No significant difference in PA at 4 wks
Annesi et al., 2013 United States [19]	Quasi-experimental, 338 (3–5 yrs)	8 wks	Daily 30 min structured GMS lessons, long-term and short-term goal setting for children	Primary/ Accelerometers	Significant difference for MVPA and VPA at 8 wks
Bellows et al., 2013 United States [20]	RCT, 201 (3–5 yrs)	18 wks	20 teacher-led activities focusing on GMS, 4 days/wk for 18 wks + 12 weeks nutrition program	Primary/ Accelerometers	No significant difference in PA at 18 wks
Bonvin et al., 2013 Switzerland [21]	Cluster RCT, 373 (2–4 yrs)	8 mths	Daily PA period, rearrangement of indoor and outdoor play spaces, $1500 for rearrangement of built environment	Primary/ Accelerometers	No significant difference in PA at 18 wks
Cardon et al., 2009 Belgium [22]	RCT, 583 (4–5 yrs)	6 wks	Four interventions: (1) play equipment, (2) markings, (3) play equipment and markings, (4) control	Primary/ Accelerometers	No significant difference in PA at 6 wks
De Bock et al., 2010 Germany [23]	RCT, 826 (4–6 yrs)	9 mths	Gym lessons (duration and frequency not specified)	Primary/ Accelerometers	Significant difference for TPA at 9 mths
De Craemer et al., 2014 Belgium [24]	RCT, 472 (4–6 yrs)	24 wks 1 yr	1 h structured lesson once/wk. Classroom activities and excursions. Rearrangement of indoor classroom play space	Primary/ Accelerometers	Significant difference for VPA during after-school hours at 1 yr
Eliakim et al., 2007 Israel [34]	Cluster RCT, 101 (5–6 yrs)	16 wks	Educational sessions with games, printed information, 45 min PA sessions 6 days/week; exercise circuit (indoors and outdoors) with mostly endurance activities, some coordination and flexibility, and additional nutrition education component	Secondary/ Pedometers	Significant difference daily steps for entire day, during school hours and after school significant difference in fitness at 16 wks
Finch et al., 2014 Australia [25]	Cluster RCT, 245 (3–5 yrs)	4 mths	Daily 20 min structured GMS session, staff role modelling, rearrangement of indoor and outdoor environment	Primary/ Pedometers	No significant difference in PA at 4 mths
Fitzgibbon et al., 2005 United States [35]	Cluster RCT, 409 (3–5 yrs)	14 wks 1 and 2 yrs	40 min education session 3 days/wk (20 min for nutrition, 20 min for aerobic activity)	Secondary/ Parent-proxy report	No significant difference in PA at 1 or 2 yrs
Fitzgibbon et al., 2006 United States [36]	Cluster RCT, 401 (3–5 yrs)	14 wks 1 and 2 yrs	40 min education session 3 days/wk (20 min for nutrition, 20 min for aerobic activity)	Secondary/ Parent-proxy report	No significant difference in PA at 1 or 2 yrs
Fitzgibbon et al., 2011 United States [37]	Cluster RCT, 190 (3–5 yr)	14 wks	20 min structured lesson related to PA, twice/wk	Secondary/ Accelerometers	Significant difference for MVPA, VPA at 14 wks
Froehlich Chow et al., 2016 Canada [26]	RCT, 69 (4.9 yrs)	48 wks	Multi-component focusing on food and physical activity	Primary/ Accelerometers	Significant difference for MVPA at 48 wks
Jones et al., 2011 Australia [27]	RCT, 97 (4.1 yrs)	20 wks	Structured 20 min activities to improve GMS 3 times/wk + teacher engagement with children during unstructured time	Primary/ Accelerometers	No significant difference in PA at 20 wks
Jones et al., 2016 Australia [28]	RCT, 150 (4.0 yrs)	6 mths	Structured 20 min activities to improve GMS 3 times/wk + teacher engagement with children during unstructured time	Primary/ Accelerometers	No significant difference in PA at mths
Nemet et al., 2011, 2013 Israel [38]	Cluster RCT, 795 (3–6 yrs)	1 yr 2 yrs	Educational sessions with games, books, printed information, 45 min PA session 6 times/wk	Secondary/ Accelerometers	Significant difference in fitness (measure of PA) at 1 and 2 yrs
O’Dwyer et al., 2013 United Kingdom [29]	Cluster RCT, 218 (3–5 yrs)	6 wks 6 mths	1 h structured active play sessions once/wk	Primary/ Accelerometers	No significant difference in PA at 6 mths
Parish et al., 2007 United States [30]	Quasi-experimental, 21 (2.6 yrs)	Unknown	2 × 30 minute play sessions/wk	Primary/ Heart rate monitors	Significant difference in heart rate (measure of PA)
Puder et al., 2011 Switzerland [31]	Cluster RCT, 421 (4–6 yrs)	1 yr 2yrs	4 × 45 min PA sessions/wk, 22 sessions on healthy nutrition, media use and sleep.	Primary/ Accelerometers	Significant difference in fitness (PA measure) at 1 yr
Reilly et al., 2006 Scotland [39]	Cluster RCT, 545 (4.2 yrs)	24 wks 1 yr	30 min enhanced PA program, 3 days/wk, posters	Secondary/ Accelerometers	No significant difference in PA at 24 wks and 1 yr
Roth et al., 2015 Germany [32]	RCT, 709 (4.7 yrs)	1 yr	5 × 30 min PA sessions/wk, PA homework cards and seasonal letters with games over the holidays	Primary/ Accelerometers	Significant difference in MVPA at 12 months
Trost et al., 2008 United States [33]	RCT, 42 (3–5 yrs)	8 wks	10 min lesson, 2 days/wk for 8 weeks, integrated PA into other key learning experiences	Primary/ Accelerometers	Significant difference in MVPA in classroom and outside for wks 7 and 8

RCT: randomized controlled trial, MVPA: moderate- to vigorous-intensity physical activity; VPA: vigorous-intensity physical activity; TPA: total physical activity; GMS: gross motor skills; wks: weeks; mths: months; yr: year; PA: physical activity; SB: sedentary behavior. PA outcome-primary: the physical activity outcome was the primary outcome for the intervention; PA outcome-secondary: the physical activity outcome was a secondary outcome for the intervention.
